# How can advocates leverage power to advance comprehensive regulation on ultra-processed foods? learning from advocate experience in Argentina

**DOI:** 10.1186/s12992-024-01069-1

**Published:** 2024-09-09

**Authors:** Simone Wahnschafft, Achim Spiller, Beatriz Andrea Graciano

**Affiliations:** 1 Research Training Group in Sustainable Food Systems, Georg-August-Universität Göttingen, Heinrich-Düker-Weg 12, 37073 Göttingen, Germany; 2 Marketing for Food and Agricultural Products, Department of Agricultural Economics and Rural Development, Georg- August-Universität Göttingen, Platz der Göttinger Sieben 5, 37073 Göttingen, Germany; 3 Free Chair of Food Sovereignty, School of Nutrition of the Faculty of Medical Sciences, Universidad de Buenos Aires, Marcelo Torcuato de Alvear 2202, Buenos Aires, Argentina

**Keywords:** Power analysis, Food governance, Ultra-processed foods (UPFs), Political economy of health

## Abstract

**Background:**

The use of corporate power to undermine public health policy processes is increasingly well understood; however, relatively little scholarship examines how advocates can leverage power to promote the successful adoption of public health policies. The objective of this paper is to explore how advocates leveraged three forms of power – structural, instrumental and discursive – to promote the passage of the Promotion of Healthy Eating Law (Ley 27,642) in Argentina, one of the most comprehensive policies to introduce mandatory front-of-package (FOP) warning labels and regulate the marketing and sales of ultra-processed foods (UPFs) adopted to date.

**Methods:**

We conducted seventeen semi-structured interviews with advocates from different sectors, including civil society, international agencies, and government. Both data collection and analysis were guided by Milsom’s conceptual framework for analyzing power in public health policymaking, and the data was analyzed using hybrid deductive and inductive thematic analysis.

**Results:**

Advocates harnessed structural power through the leveraging of revolving doors, informal alliances, and formal coalitions, enabling them to convene discussion spaces with decision-makers, make strategic use of limited resources, and cultivate the diverse expertise (e.g., research, nutrition science, advocacy, law, political science, activism and communications) needed to support the law through different phases of the policy process. Advocates wielded instrumental power by amassing an armada of localized evidence to promote robust policy design, building technical literacy amongst themselves and decision-makers, and exposing conflicts of interest to harness public pressure. Advocates exercised discursive power by adopting a rights-based discourse, including of children and adolescents and of consumers to transparent information, which enabled advocates to foster a favorable perception of the law amongst both decision-makers and the public. Key contextual enablers include a political window of opportunity, the COVID-19 pandemic, and the ability to learn from the regional precedent of similar policies.

**Conclusions:**

Public health policymaking, particularly when encroaching upon corporate interests, is characterized by stark imbalances of power that hinder policy decisions. The strategies identified in the case of Argentina provide important insights as to how advocates might harness and exercise structural, instrumental, and discursive power to counter corporate influence and promote the successful adoption of comprehensive UPF regulation.

**Supplementary Information:**

The online version contains supplementary material available at 10.1186/s12992-024-01069-1.

## RESUMEN

**Antecedentes:**

El uso del poder corporativo para socavar los procesos de políticas de salud pública se comprende cada vez mejor; sin embargo, relativamente pocos estudios examinan cómo los defensores pueden aprovechar el poder para promover la adopción exitosa de políticas de salud pública. El objetivo de este artículo es explorar cómo sus defensores aprovecharon tres formas de poder – estructural, instrumental y discursivo – para promover la aprobación de la Ley de Promoción de la Alimentación Saludable (Ley 27.642) en Argentina -una de las políticas más integrales adoptadas hasta la fecha- que establece la obligatoriedad del etiquetado frontal de advertencias en alimentos y bebidas envasados y regula la comercialización y venta de productos comestibles y bebibles ultraprocesados (PUP).

**Métodos:**

Realizamos diecisiete entrevistas semiestructuradas con defensores de diferentes sectores, incluyendo sociedad civil, agencias internacionales y gobierno. Tanto la recolección como el análisis de datos se guiaron por el marco conceptual de Milsom para analizar el poder en la formulación de políticas de salud pública, y los datos se analizaron mediante un análisis temático híbrido deductivo e inductivo.

**Resultados:**

Los defensores sacaron provecho del poder estructural mediante el uso de puertas giratorias, alianzas informales y coaliciones formales, lo que les permitió generar espacios de discusión con los tomadores de decisiones, hacer uso estratégico de recursos limitados y profundizar la experticia diversa (por ejemplo, en investigación, ciencia de la nutrición, abogacía, derecho, ciencias políticas, activismo y comunicación) necesaria para apoyar la ley a través de las diferentes fases del proceso político. Los defensores ejercieron poder instrumental al acumular abundante evidencia local para promover un diseño de políticas sólidas, construir conocimientos técnicos entre ellos y los tomadores de decisiones, y exponer conflictos de intereses para ejercer presión pública. Los defensores ejercieron poder discursivo al adoptar un discurso con enfoque de derechos -incluyendo el de niños, niñas y adolescentes, y el de los consumidores a acceder a una información transparente-, lo que les permitió fomentar una percepción favorable hacia la ley tanto entre los tomadores de decisiones como entre el público. Los facilitadores contextuales clave incluyen una ventana de oportunidad política, la pandemia de COVID-19 y la capacidad de aprender de la experiencia regional en políticas similares.

**Conclusiones:**

La formulación de políticas de salud pública, particularmente cuando menoscaban los intereses corporativos, se caracteriza por marcados desequilibrios de poder que obstaculizan las decisiones políticas. Las estrategias identificadas en el caso de Argentina brindan ideas importantes sobre cómo los defensores podrían aprovechar y ejercer el poder estructural, instrumental y discursivo para contrarrestar la influencia corporativa y promover la adopción exitosa de una regulación integral de PUP.

## Background

Since their emergence in the mid-20th century, ultra-processed foods (UPFs) have rapidly taken center stage in changing dietary patterns around the world. Including such products as packaged sweet and savory snacks and sugary drinks, UPFs are generally energy dense, high in dietary components with health-harming effects (e.g., sodium, sugar, saturated fats, and trans-fatty acids), and laden with cosmetic food additives and/or other industrial ingredients, many with unknown health effects [1]. In some high-income countries (HICs), UPFs already account for 50–60% of daily energy intake, and low- and middle-income countries (LMICs) are following suit [2]. In fact, annual sales growth of UPFs in middle-income countries (MICs) already far surpass that of HICs [3] and sales volume are anticipated to reach HIC levels by 2024 [4]. Chronic consumption of UPFs is associated with higher risk for a suite of chronic diseases, including obesity, cardiovascular disease, cancer, type II diabetes, asthma, and depression [5]. The agro-industrial complex needed to support the cultivation of basic ingredients, manufacturing and mass distribution of UPFs, increasingly at the expense of traditional, minimally processed foods, contributes to a host of adverse environmental outcomes, such as land degradation, climate change, and agrobiodiversity loss [6, 7].

For the past two decades, scholars have sought to identify explanations for shifting dietary patterns and the consequent burden of chronic disease. For example, the ‘nutrition transition’ emerged in the early 2000s as a prevailing model to explain shifts from traditional dietary patterns towards ‘Western’ diets characterized by high UPF consumption, pointing to variables like economic development, modernization, urbanization, and increased wealth as drivers of the transition [8, 9]. More recently, scholars have stressed the importance of adopting a political economy approach, placing actors and the power relationships between them at the heart of analysis, to examine how power has been consolidated amongst national and transnational food and beverage companies to favor the widespread availability, affordability and accessibility of UPFs [10–12]. Such analyses have pointed to factors such as trade and investment liberalization [13, 14], increasing market concentration [15], and the rise of hybrid food governance arrangements, such as public-private partnerships [16, 17], as key drivers of corporate power in food governance. This consolidation of power is not unique to food: in 2018, of the world’s largest economies, 29 were countries and 71 were corporations [18]. The commercial determinants of health (CDoH) have emerged as an increasingly prominent area of research and discourse to call attention to this formidable influence corporations now wield in shaping health outcomes [19].

This emerging body of literature on corporate power in food governance has also increasingly been called upon to explain why, despite calls to action on the need for regulatory approaches [16], and guidance on policies needed to ameliorate unhealthy food environments characterized by widespread UPFs [20–23], policy responses to date have been glaringly inadequate [12]. Indeed, country governments to date have predominantly favored the adoption of interventions targeting individual behavior change, such as education (75% of countries) and media campaigns (61%) over regulatory actions on UPFs, such as front-of-pack (FOP) labelling schemes (25%), and restrictions on child-directed marketing (31%) [24]. Corporate political activity (CPA), referring to industry efforts to influence public policy, research and practice, plays a major role in preventing, weakening, or delaying regulatory approaches for improving food environments [25]. Researchers have increasingly sought to catalogue and monitor CPA in policy processes to regulate UPFs [26], as well as on other health-harming commodities like breastmilk substitutes [27] and alcohol [28], around the world [29–31].

Despite pervasive challenges to regulate UPF consumption in the face of contemporary corporate power dynamics, a small precedent of success has been set by a handful of countries in the adoption of UPF regulatory policies. Most of these countries are in Latin America, where UPF consumption has grown exponentially in the 21st century [32], alongside the prevalence of diet-related disease morbidity and mortality [33]. In 2016, Chile became the first country worldwide to jointly introduce a package of three policy measures to address unhealthy food environments: (1) mandatory FOP warning labels on UPFs, (2) restrictions on child-directed marketing of UPFs, and (3) a ban on UPF sales in schools [34]. This approach to bundle, or package, several policy measures into one intervention aligns with international guidance to comprehensively address multiple drivers of unhealthy food environments [21, 22]. Other countries in the region have since followed suit to adopt similar policies, though with quite variable outcomes in terms of policy design (e.g., type of FOP label, types of food marketing restricted), stringency (e.g., nutrient profile model specifications), and comprehensiveness of policies adopted (i.e., FOP labels packaged with additional measures or labels alone) [35]. CPA to prevent, delay, or weaken regulatory action has been well-documented as a major challenge through these policy processes, including in Chile [36], Colombia [37], Mexico [38], Uruguay [39], and Brazil [40].

While a growing scholarship has been dedicated to de-mystifying the ins and outs of how CPA – or the ‘corporate playbook’ – is used to protect industry interests and stymie public health policy, comparatively little scholarship has been devoted to examining how public health advocates can counter this activity and successfully promote the adoption of public health regulatory policies. Those studies that have been conducted predominantly examine the role and strategies of advocates, particularly within civil society, to counter industry interference and advance regulation in the realm of tobacco control [41–46], as well as sugary drink taxes [47–51], and health-harming commodities more broadly [52–54]. A small body of studies were identified that sought to learn from the experience of advocates in UPF regulation in Latin America to date, including in Chile [34, 55], Mexico [55], Brazil [55], Uruguay [39], and Peru [56]. However, only a few of these studies [49, 51, 52, 56] directly engage with concepts and/or empirical analyses of power in these policy processes. Power analysis is an important tool to build a nuanced understanding of how and why the strategies employed by different stakeholders to further their interests do (or do not) result in desired outcomes [10]. Empirical analyses of power in real-world policy experiences have been identified as a key gap in research in public health governance [57, 58], and are sorely needed to develop a ‘public health playbook’ of strategies to counter and proactively minimize corporate influence [59]. The aim of this paper therefore is to examine how advocates were able to exercise power to promote the recent adoption of the food environment policy package, the Promotion of Healthy Eating Law (Ley 27,642), in Argentina. The remainder of this section provides an overview of key concepts and trends in UPF regulation, as well as an overview of the Argentinian regulation, before delving into the methods.

### Regulating ultra-processed foods: key concepts and trends

Though the concept first emerged in the 1980s [60], the term ‘ultra-processed foods’ began to rise to prominence in 2009 with the emergence of the NOVA classification, a system that categorizes food products across four different levels (e.g., (1) unprocessed or minimally processed foods, (2) processed culinary ingredients, (3) processed foods, and (4) ultra-processed foods) according to the type, intensity, and purpose of food processing [61]. Within this system, UPFs refer to those foods with the highest level of processing, I.e., those that have ‘undergone intense industrial physical, chemical, or biological processes (e.g., hydrogenation, moulding, extruding, preprocessing by frying) or that contains industrial substances not usually found in domestic kitchens (e.g., maltodextrin, hydrogenated oils, or modified starches), cosmetic additives (e.g., dyes, emulsifiers, artificial sweeteners), or flavouring agents’ [61, 62].

While the most prominently used definition of UPFs hinges on the level of processing, several countries have begun to move forward on UPF regulation using a ‘nutrient based’ approach rather than one based on the level of processing. That is to say that these policies aim to regulate the labelling, marketing, and sales of UPFs based on their level of ‘critical nutrients,’ such as added sugars, sodium, saturated fats, and trans fats. This approach continues to be subject to debate, as the nutrient-based approach to regulation generally does not take into account several components of UPFs with detrimental effects on health, such as artificial sweeteners, colorants, preservatives, thickeners, and emulsifiers [62].

This nutrient-based approach underpins the UPF regulatory approaches that have emerged in several countries in Latin America over the past decade, particularly with the adoption of mandatory FOP warning labels and, in some cases, accompanying marketing and sales restrictions, that began in Chile and that have since been adopted in Peru, Uruguay, Mexico, Colombia, and Venezuela [35]. While these policies follow a similar general approach, they are characterized by important nuances in policy design that enable some to be considered more robust than others from a regulatory perspective.

First, with regard to FOP labelling schemes, these nuances include aspects such as the mandated size of the label and the use of contrasting background devices to improve the salience of the label on product packaging [35]. Which nutrients are to be labelled is also a key issue, with some countries expanding beyond the scope of those nutrients such as sugars, sodium, and fats to also label artificial sweeteners and caffeine [35]. Another important aspect is the phrasing of the warning label itself, with some countries moving towards the use of the stronger “excess in” rather than “high in” phrasing [35]. The nutrient profile model (NPM) used to define the threshold of the label is also critical, with the NPM developed by the Pan-American Health Organization (PAHO) [63] considered to be best practice for the region of the Americas [35]. Finally, with regard to those measures that accompany FOP labels, important differences are present in the scope of marketing restrictions, with some countries moving beyond those focused solely on child-directed marketing to also include restrictions on health or nutrition claims, endorsements, and other persuasive elements for products with warning labels [35]. These nuances are further explored in the following section, which lays out the key tenets achieved in the Argentinian regulation, as well as how they compare to other regulatory precedents in the region.

### The Promotion of Healthy Eating Law

In keeping with regional trends, sales and consumption of UPFs in Argentina have increased throughout the 21st century, now constituting nearly 26% of daily energy intake [64]. The Promotion of Healthy Eating Law (Ley 27,642) [65], also commonly referred to as the ‘front-of-package nutrition labelling law’ (*ley de etiquetado frontal)* was adopted in 2021 to regulate the labelling, marketing, and sale of UPFs. Since its passage, the law has been deemed to be one of the strongest and most comprehensive food policy laws globally due to several aspects of the policy design, expanded upon below [66].

First, the FOP labelling system adopted follows the latest regional guidance [67] and nationally generated evidence [68–72] on the most effective design for decreasing UPF consumption. Specifically, the law includes the mandatory introduction of black octagonal warning labels, which are to be added to the front of UPFs deemed in “excess” of sugars, total fats, saturated fats, sodium, and/or calories, taking the phrasing of the labels further than those of several others in the region, including Chile, Colombia and Peru [35]. In addition, the labelling system adopted by the law includes two pre-cautionary labels related to the presence of caffeine and sweeteners in UPFs to be avoided by children and prohibits the use of health claims on products containing at least one warning label, both of which are otherwise only addressed in UPF regulation in Mexico [35]. The Argentinian regulation has also been identified as the strongest in the region with regard to the mandated size of the warning labels on product packaging, meeting the PAHO recommendation that all labels together should occupy at least 30% of the main product display panel [35, 67] Finally, the adoption of PAHO’s NPM as the basis of the warning labels in Argentina can be regarded as a critical success, which has otherwise only been achieved in Mexico [35]. Other countries in the region, including Uruguay, Peru, and Venezuela, sought to adopt the PAHO NPM, but ultimately adopted less stringent systems [35, 39].

Another strength of the policy is the presence and scope of accompanying measures included in the policy package related to marketing, namely the prohibition of advertising, marketing, and sponsorship of all products with at least one warning label towards children and adolescents, including the use of children’s characters, cartoons, celebrities, athletes, influencers and more. These restrictions apply both to product packaging and advertising in traditional and digital media. Finally, the law stands out for the comprehensiveness of included measures directed towards improving food environments. For instance, the law prohibits the sale, offering, and marketing of products with at least one warning label on school premises and introduces mandatory nutrition education at all levels of mandatory education. Public procurement, such as that which would affect social support programs, is also affected by the law, obligating the prioritization of products without warning labels when comparing procurement offers.

There are also several notable aspects of the law related to the policy process itself that distinguish it as a robust policy case. For example, the fact that UPF regulation in Argentina was ultimately adopted through the Legislative branch in the form of a law, rather than the Executive branch in the form of a decree, is important, as it offers the policy a greater degree of protection from changing political forces. This can be distinguished from, for instance, the labelling policy adopted in Uruguay, which was adopted as a decree through the Executive branch and became subject to several changes throughout the policy adoption process that eroded the scope of the initial proposal [39]. The Argentinian case is also notable for the degree of support with which it was passed through both chambers of the National Congress: first in the Senate (64 votes in favor, 3 votes against, 0 abstentions) and then the Chamber of Deputies (200 votes in favor, 22 votes against, 16 abstentions). This degree of support is notable in a country where industry holds high financial and political power. Agriculture and agro-industry together constitute one of the most important industries in Argentina, accounting for an estimated 8% of GDP, 20% of employment and 54% of exports [73]. In addition, food and beverage processing accounts for over half of agro-industrial production [73]. Sugar is also an important agricultural product, particularly in the northwest provinces of Salta, Jujuy, and Tucumán, where politicians have previously leveraged their power to prevent the introduction of regulatory policies, such as a sugary drinks tax [74]. A final notable facet regarding the politics of UPF regulation in Argentina is that of trade, as Argentina is a member of the Southern Common Market (MERCOSUR), a regional trade agreement between Argentina, Brazil, Uruguay and Paraguay. Argentina’s membership in MERCOSUR is relevant in the context of a growing literature on the role of trade agreements in hindering regulation of health-harming commodities [75–80] and emerging evidence on the strategy used by food industry actors to hinder advancement on national front-of-package labelling policies in the bloc by insisting on the need for a regional (I.e., bloc-wide) regulatory approach to FOP labelling to avoid threats to international trade [39, 40].

## Methods

### Conceptual framework

**Fig. 1:**
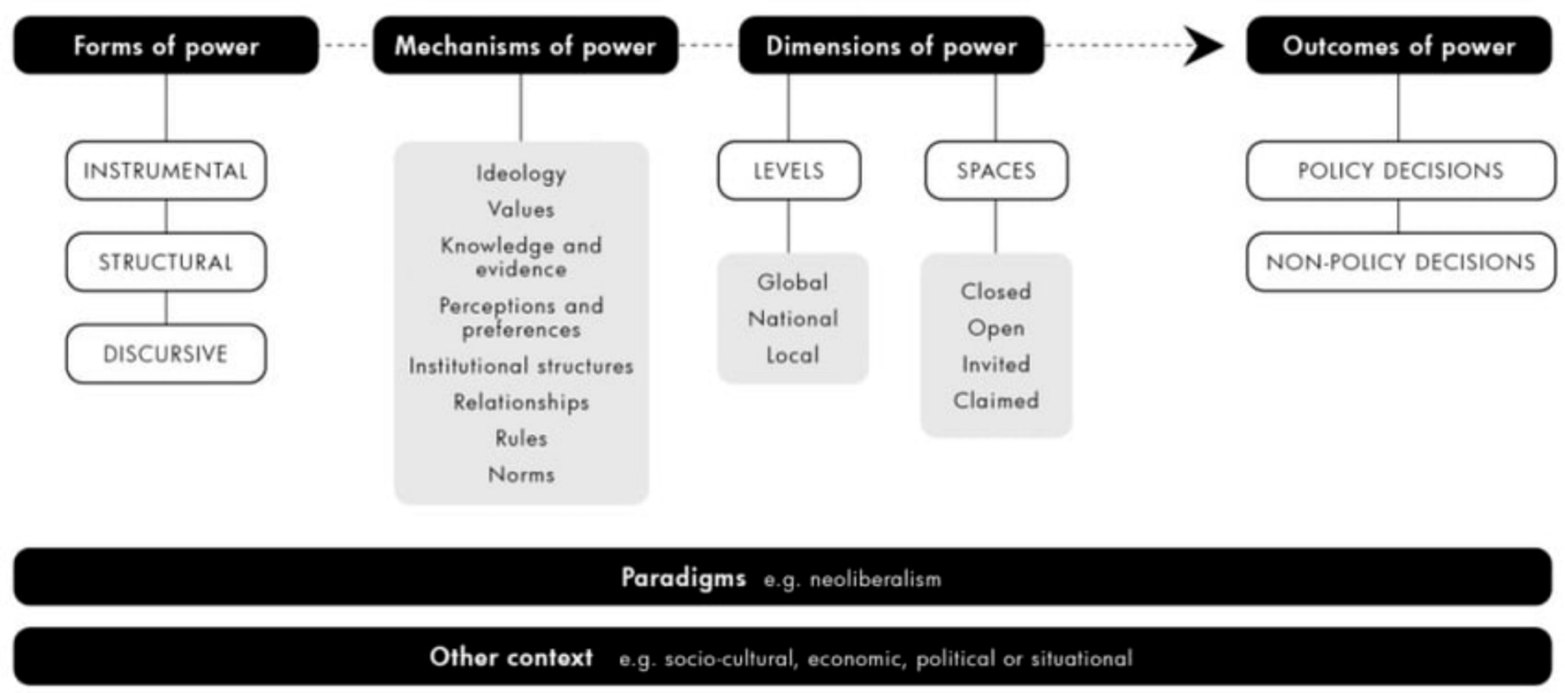
Milsom’s conceptual framework for analyzing power in public health policymaking

We utilized Milsom’s conceptual framework for analyzing power in public health policymaking [78] to guide the conduct and analysis of in-depth, semi-structured interviews with identified advocates of the law leading up to its adoption in 2021. Drawing upon a synthesis of existing political economy and power frameworks –most notably Fuchs and Lederer’s framework on business power in global governance [81], Lukes’ Three Dimensions of Power [82], the ‘Three Is’ framework [83–86], and Gaventa’s power cube [87] – this framework details how actors can harness power to either successfully promote health policy decisions or hinder them, delineating three key forms of power that actors can exercise: instrumental, structural and discursive (see Fig. 1).

The former, which is usually the most visible, refers to direct influence actors can exercise to advance their interests through their actions. The latter two forms are generally more hidden, with structural power encompassing aspects like agenda-setting and rule-setting power, as well as the capacity to secure a ‘seat at the table’ in decision-making spaces. Finally, discursive power refers predominantly to how actors influence the way in which issues are discussed (e.g., framing) in decision-making spaces. These forms of power can be exercised through several mechanisms, such as existing institutional structures, the use of knowledge and evidence, and the cultivation of relationships, and can be exercised across different dimensions, including at local, national or global levels, and in spaces that are either closed, open, invited or claimed. Though presented separately, these forms, mechanisms and dimensions of power are not necessarily independent, and rather can interact. Together, these aspects of power, and respective policy (non-)decisions sit within political, economic, socio-cultural or situational contexts that either hinder or enable the exercise of power. Milsom and colleagues recently applied this framework to examine how corporations exercise power through the international trade regime to hinder policy decision-making on UPFs [78–80]. In this paper, we turn rather to the context of a successful policy decision (I.e., policy adoption) to examine, from their own perspective, how advocates were able to leverage power to advance the passage of the Promotion of Healthy Eating Law (Ley 27,642).

### Identifying policy advocates

We used three types of documents to identify policy advocates for interviews: media articles, press releases, and grey literature (I.e., reports). We devised the following set of search terms to identify and collate these documents: ‘etiquetado frontal,’ ‘rotulado’, ‘promoción de alimentación saludable,’ ‘restricción de la comercialización’. First, we applied these terms to a systematic search of nine media outlets that represent a range of political leanings from left-wing to conservative, as identified from the BBC media guide in Argentina [88] (see Supplementary Materials, Table A1). Then, through an initial screening of these documents, we identified websites of stakeholder organizations that were identified as working to advance the law through the policy process, which we searched with the same terms for press releases and grey literature relevant to the analysis (see Supplementary Materials, Table A2. We then screened all sources identified using the search terms to ensure they met the following inclusion criteria: (1) focused on Ley 27,642 or reference to agenda-setting on policy measures ultimately included in the law in Argentina (I.e., FOP labelling, marketing restrictions, and/or improving school food environments); (2) included a description of policy process milestone(s) (e.g., meetings held, stakeholders involved, actions and decisions taken, industry arguments and counter-arguments, etc.); (3) were published in English or Spanish; and (4) were published up until the adoption of the law in 2021 (see Supplementary Materials Table A3). We then used these sources to construct a database of relevant organizations and individuals who were highlighted as advocates of the policy, subsequently inviting a first set of advocates from civil society to participate in the study by email. Email outreach included a description of the project aims, a document with additional information for participants, and a copy of the consent form. These outreach materials were reviewed and approved, alongside the interview guide, by the Ethics Committee at Georg-August-Universität Göttingen prior to data collection. This phase of the research was also used to construct an overview of key stakeholders and milestones in the policy process, both of which are available in the Supplementary Materials(Table A4, Table A5).

### Conducting interviews

We conducted seventeen policy advocate interviews between November 2022 and April 2023, including stakeholders with roles across civil society, academia, international development agencies, and both the Executive and Legislative branches of government (see Table 1). While we conducted our initial outreach to civil society stakeholders, we decided not to limit our definition of ‘advocate’ to civil society stakeholders alone, but rather to allow participants to define who would be important stakeholders to speak to who fit this description. We accomplished this by way of snowball sampling, as each participant was asked at the end of the interview to identify additional advocates they would recommend be included in the study. We followed recently published guidance on the principle of determining sample size for saturation (I.e., the point at which little or no relevant new codes and/or categories are to be found in data) a priori in qualitative research, which is identified as 9–17 interviews for studies with homogenous populations and narrow research objectives, as in this case [89]. In addition, we ensured that we continued with interviews until we continued to hear the same names recommended and no new themes emerged during interviews. Based on indicated participant preferences, interviews were conducted either directly in English, or in Spanish with the support of simultaneous interpretation. Apart from one interview conducted with two policy advocates simultaneously, participants were interviewed alone. Interviews were conducted in person in Buenos Aires or online using teleconferencing, again depending on participant preference. Participants were required to provide informed consent that they agreed to have the interview recorded. Recordings were transcribed in the original language of the participant’s interview and, in the case of Spanish transcriptions, translated into English with the support of a professional translation service in Argentina.

**Table 1:** Number and type of policy advocates interviewed

Stakeholder type*	Number of policy advocates
Civil society	6
Professional nutrition organization	3
Academia	1
International development agency	2
Executive branch (Ministry of Health)	2
Legislative branch (Advisor, Legislator)	3
Total	**17**

*Refers to identified main role during the policy process, not necessarily current role

Informed by Milsom’s conceptual framework, the interview guide first prompted participants to reflect on both the perceived challenges and key strategies to their work in advocating for the law. This line of questioning often brought participants naturally to the topic of grappling with corporate influence; however, if not, this topic was then broached with direct questions on perceived power asymmetries and intervention points upon CPA. Participants were also asked to reflect on lessons learned from the policy process and contextual factors they believed enabled the law to advance. See **Supplementary Materials**,** Annex 2** for the interview guide.

### Analyzing data

The interview transcripts were analyzed using thematic analysis, following a hybrid deductive and inductive approach [90, 91]. First, the primary researcher (S.W.) developed a codebook based on Milsom’s conceptual framework, applying it to the analysis of the interview transcripts in MAXQDA software. Throughout this process, additional codes were developed inductively to capture key themes, including advocate challenges, strategies, lessons learned, contextual enablers, and reflections on power and outcomes through the policy process. Then, a second researcher (B.A.G.) coded two of the transcripts using both the deductive and inductive codes, which were then reviewed by both researchers to resolve any discrepancies in coding. Following a brief overview of the policy process, the findings are presented in the results section according to the three forms of power of Milsom’s conceptual framework, with relevant information related to the mechanisms and dimensions of power integrated throughout each of the three sections. Additional sections on contextual enablers and reflections on outcomes of the policy process are also included. Some data sourced from the document review is integrated throughout the results to provide additional context to the analysis of the interview data.

## Results

### Overview of the policy process

Proposals on FOP warning labelling in Argentina began to emerge in 2016 [92], the same year in which Chile officially implemented its law on food labelling and advertising (Ley 20,606) and PAHO published its NPM to define limits for critical nutrients in UPFs. As one advocate emphasized, the latter was particularly impactful for the policy process in Argentina:

*“It’s a very small book that was very revolutionary. In the sense that it paved the way*,* in all countries*,* to regulate. That is the main precedent.” [Advocate*,* International Development Agency]*.

As the topic began to gain momentum in Argentina, two parallel approaches emerged to move forward on FOP labelling: one through the Executive branch and the other through the Legislative branch. The former was developed through the convening of an inter-ministerial working group beginning in 2018, led by the Ministry of Health and in partnership with the Ministry of Agriculture, Livestock and Fisheries and the Ministry of Productive Development. This inter-ministerial initiative emphasized the need to work with stakeholders from different sectors throughout the development of the proposal, including civil society, academia, professional nutrition organizations, and the food and beverage industry [93]. This initiative also sought to advance on labelling through changes agreed upon both within the National Food Commission (CONAL), which oversees the implementation of the Argentine Food Code, and with other members of MERCOSUR [94]. Following several changes to the proposal made between 2018 and 2020, in part due to a change in the government administration in 2019, the Executive branch reached a finalized proposal, which it presented in meetings with both CONAL [95] and MERCOSUR [96] around the same time that the bill began to be debated in the National Congress.

The latter approach, which was ultimately passed into law, was a bill proposed in the Senate in early October 2020. This bill was reached through a negotiation process that unified 15 different bills drafted by legislators from different political parties in the years preceding to address unhealthy food environments [97]. Upon receiving a positive opinion from the internal commissions of Health and of Industry, the bill was swiftly given half sanction by the Senate later the same month, promptly moving to the Chamber of Deputies for consideration by relevant internal commissions. The bill was then assigned to be debated by six commissions, though this was later reduced to four: General Legislation; Social Action and Public Health; Consumer Defense User and Competition; and Industry. Almost a year after the half-sanction in the Senate, and following the positive opinion given by the four commissions in July, the bill was passed into law by the Chamber of Deputies in October 2021.

### Contextual enablers

Advocates pointed to several contextual factors that shaped the successful adoption of the law. First, the COVID-19 pandemic opened a window of opportunity by elevating the protection of public health as a priority value both amongst the public and decision-makers, as well as the legitimacy of the public sector as an entity to intervene upon the private sector:

*“…the state was recognized*,* at the moment*,* as an actor that could guarantee our health; so*,* it was also related to this bill. If we let the market act independently and autonomously*,* all factories would be open*,* all enterprises would be open*,* and the virus would have spread more and increased the rate of deaths and everything. And people were scared at the time*,* so the state was seen as a positive influence in society*,* which has changed now.” [Advocate*,* Advisor to Legislator]*.

The COVID-19 pandemic, particularly during the lockdown that characterized the debate of the law in the Senate, was also identified as an important factor that shifted access to discussion spaces with decision-makers throughout the process. For instance, one advocate in civil society noted that the shift to online communication helped their organization, which was newer and characterized by few resources, access discussion spaces where the law was being debated, as well as convene spaces to bring together advocates and legislators to discuss the topic. Another advocate highlighted that the virtual nature of policymaking at this time may have also mitigated lobbying and heightened transparency in the early stages of the legislative process:

*“…the legislators were at home*,* in their provinces. It was not possible for the industry to visit them in their offices. And there are things that are not going to be negotiated via Zoom…Personally*,* I think the pandemic helped make the committee’s discussion transparent*,* it’s posted on YouTube. There weren’t any twists*,* lobbying*,* or at least it was less common*,* because people could not travel through the streets here. We had a very strict quarantine.” [Advocate*,* International Development Agency]*.

In general, advocates noted that they were readily invited, alongside industry and other stakeholders, to participate in spaces that debated the policy in both the Executive and Legislative branches, enabling them to share data and arguments with decision-makers, with the only notable exception related to discussions undertaken at the level of MERCOSUR.

Advocates also pointed to several political factors that shifted perceptions of the bill in Congress, including shortened ideological distances in Congress following the change in administration in 2019 and the fact that FOP labelling laws passed in other countries in the region were adopted by governments that came from similar ideological perspectives as the two major coalitions in Congress at the time. The latter lent the bill a degree of credibility as an innovative and politically viable measure:

*“…the previous experience in other countries was helpful because they leaned more towards the center-right*,* Chile*,* for example; and my coalition*,* [Party A]*,* also center-right*,* saw them as an inspiration. And if Chile was doing this*,* then it was a modern dynamic and innovative proposal and not some old-fashioned policy. But we also saw the experience in Uruguay*,* which is center-left*,* so [Party B] could learn from them too. So*,* we had this context.” [Advocate*,* Advisor to Legislator]*.

Another political factor that enabled the law’s successful passage was its bi-partisan support in Congress. This support was due in part to the fact that the bill that was ultimately debated in Congress was reached through the negotiation process that unified previous bills drafted by legislators from different political parties in the years preceding. This political neutrality was also further reinforced by the fact that the unified bill was proposed by two Senators who came from the same province but each from a different political party that constituted the two main coalitions at the time.

*“The fact that we managed to present a bill that was not affiliated to any political party is very important because it allowed us to receive support from both parties without any political divide.” [Advocate*,* Civil Society]*.

### Structural power

#### Convening spaces and knowledge exchange

As early momentum began to build on UPF regulation in Argentina, advocates were able to capitalize on growing attention to the topic to elevate it on the legislative agenda by organizing a series of conferences, beginning in 2016 and continuing through the legislative debate of the law [92, 98–101], which convened stakeholders across civil society, the Executive branch (e.g., national ministries), the Legislative branch, academia, and professional nutrition organizations, amongst others. These spaces were convened via a partnership of international agencies, led by PAHO. A key activity facilitated by advocates throughout these meetings was to invite champions of UPF regulations passed in other countries in the region, beginning with representatives from Chile, and proceeding with those from Uruguay, Mexico, Peru, and Colombia. Inviting stakeholders to learn from regional precedents was not only advantageous for building political momentum, but for learning from previous experiences to foresee challenges, such as CPA, that would ensue as the proposal for regulation began to gain ground:

*“It was like a regional training*,* not only were we debating this*,* but other countries as well; so*,* before Argentina*,* also other countries went along*,* Perú went along*,* Uruguay went along*,* then Mexico went along*,* and each country benefiting from the previous experience of other countries. So*,* when we learned from the experience in Chile*,* we knew how the food industry would react*,* what their strategies would be…” [Advocate*,* Academia]*.

### Revolving doors

The ability to convene these spaces with decision-makers was enabled in part by existing relationships advocates held, some of which were cultivated through prior health policy processes, including tobacco control and regulation on sodium content in foods. Several participants pointed to the importance of ‘revolving doors’ in this respect, referring to the importance of seasoned advocates who either held multiple roles at one time or changed roles across sectors during the policy process, bringing their knowledge, expertise, and networks with them to new positions:

*“We have the same people in different organizations*,* for instance*,* I mentioned three that I belong to*,* and you take this to the agenda of the organization.” [Advocate*,* Academia]*.

The most commonly noted shifts between sectors that advocates highlighted were between civil society, international development agencies, and the Ministry of Health; however, specific reference to these shifts is omitted here for anonymity purposes.

### Informal alliances

Informal alliances were identified as key to overcoming roadblocks faced by any one organization or sector. This emerged, for instance, in the context of the tension between the Executive and Legislative proposals that were developing simultaneously in the years leading up to the official proposal of the bill in Congress. Both proponents and opponents sought to leverage these two institutional approaches to UPF regulation to their respective advantage to either promote or hinder the law from advancing. For example, the Coordinator of Food Product Industries (COPAL), an umbrella entity representing the interests of the food and beverage industry, supported the proposal furthered by the Executive branch rather than the bill. Advocates identified this as CPA, as the Executive proposal was inferior to the Legislative approach for several reasons, including higher susceptibility to shifts in political climate, lower alignment with best practice in both scope and stringency of proposed regulation, and the fact that pursuing a regional agreement with other members of MERCOSUR would significantly delay the process, as well as place the debate in an arena in which industry and trade interests were paramount. Indeed, stakeholders representing industry interests often used their resources to disseminate the argument that Argentina was not *allowed* to regulate without agreement at the level of MERCOSUR to stifle support for the bill in Congress:

*“…they started to say that we could not move forward with the law because it went against our integration within the MERCOSUR*,* even though legally it was not the case. But they still claimed this*,* which confused legislators.” [Advocate*,* Advisor to Legislator]*.

Advocates leveraged structural power in the face of this challenge through informal alliances with advocates across different positions. Particularly for those advocates facing institutional constraints, such as those positioned within the Executive branch, the importance of informal alliances was key:

*“…the best option was always the bill*,* and even then*,* we knew that a bill would not involve the Executive Branch we worked at. So*,* it was our priority to ally with the organizations that would work closely with deputies and senators to try and convince them to be in favor of this policy.” [Advocate*,* Ministry of Health]*.

Within civil society, informal alliances also allowed advocates to collectively permeate spaces where misinformation was spread to decision-makers to undermine the bill. This was the case, for instance, for some conferences held by professional nutrition organizations with known conflicts of interest. As one advocate explained:

*“…thankfully we were able to work together with other civil society organizations. Whenever one was not able to participate*,* the others would be there to support them. So*,* in certain meetings they would say: “Let’s invite [Organization A] because they aren’t as antagonistic as [Organization B]”. But*,* thanks to our alliance with other organizations*,* we were always able to speak for each other*,* to empower each other.” [Advocate*,* Professional Nutrition Organization]*.

### Coalition building

Formal alliances, in the form of coalitions [102–104] also helped advocates harness structural power through different stages of the policy process. For example, the resources developed by the National Coalition to Prevent Childhood Obesity [105], formed in 2017 with support from UNICEF Argentina [106], lent a collective voice of legitimacy to the positions advocated by a handful of organizations working at the forefront of the process:

*“…that was also a very important support because it is different to say to the legislature “[Organization A] has this policy brief”*,* no*,* this policy brief is supported by more than 40 organizations representative from all the country. That is*,* well*,* the legislators paid a lot of attention.” [Advocate*,* Civil Society]*.

During the legislative debate, formal coordination across civil society organizations also became an important strategy for building capacity both within and across organizations. This was particularly the case when five civil society organizations collectively secured a grant funded by Bloomberg Philanthropies and managed by the Global Health Advocacy Incubator (GHAI). With the support of the grant, these organizations were able to expand and diversify their own activities in support of the law, as well as to organize activities collectively. The latter was identified as a challenge by several advocates, as it required novel coordination across organizations with different reputations, approaches, and leadership structures; however, it was also noted as a key strategy to harness collective action and effectively counter corporate power:

*“I mean*,* the inequality of arms was evident from the beginning: the industry has all the means for advertising*,* for paying nutritionists*,* to go in the media to demonize the law*,* and we had nothing. But this influx of money through GHAI to the organization allowed us to counteract that.” [Advocate*,* Academia]*.

### Instrumental power

#### Wielding evidence

The generation and dissemination of knowledge and evidence was one of the key activities advocates led to influence the policy process (see Table 2 for a summary of key studies). For instance, a series of studies led by the research-oriented civil society organization, the Inter-American Heart Foundation - Argentina (FIC-Argentina) in 2015–2018 demonstrated the widespread exposure of children and adolescents to different forms of UPF marketing in Argentina [107–109], elevating the issue of the need for regulation. Along a similar vein, two studies conducted by the Ministry of Health – the 4th National Survey of Risk Factors (2018) [110] and the 2nd National Survey of Nutrition and Health (2019) [111] - provided updated evidence on UPF consumption trends in Argentina and the burden of diet-related chronic disease to underscore the extent of the challenge in Argentina.

**Table 2:** Knowledge and evidence documents generated by policy advocates in Argentina

Document	Relevance to policy process	Year of publication	Author(s)*
- Food advertising aimed at boys and girls on Argentine TV	Examine prevalence of and exposure to UPF marketing amongst children and adolescents	2015	FIC
- Marketing techniques aimed at boys and girls in processed food packaging in Argentina		2017	
- Food advertising on Argentinean television: are ultra-processed foods in the lead?		2018	
- Exposure of boys, girls and adolescents to digital marketing of food and beverages in Argentina		2021	UNICEF
- 4th National Survey of Risk Factors	Examine the nutrition and prevalence of diet-related diseases and associated risk factors in the population	2018	MOH
- Argentina National Survey of Nutrition and Health, 2018–2019 (ENNyS 2)		2019	
- Sugary drinks in Argentina: burden of disease and impact of health interventions		2020	IECS
- Lessons learned from tobacco control: court decisions that ratify public health policies	Develop an understanding of the political and regulatory landscape	2020	FIC
- Front warning labelling bill: economic arguments that support it		2020	
- Regulatory mapping: front food labelling		2020	FIC and IDEC
- Front food labelling in Argentina and Brazil: legal barriers and facilitators		2020	
- Conflict of interest and interference of the food industry in the design of healthy eating policies		2020	Various
- Evaluation of the performance of the front of package warning labelling compared to other models in Argentina	Supporting evidence for effective policy design	2020	MOH
- Analysis of the level of concordance of nutrient profile systems with the Dietary Guidelines for the Argentine Population		2020	
- Evaluation of nutrient profile systems Nutritional for the definition of a front of package labelling policy in Argentina		2020	FIC
- Survey to evaluate the influence of three front of package labelling systems in the perception of healthiness and the purchase intention of certain products - Opinion survey on front labelling of warnings in food and drinks		2021	

*FIC = Inter-American Heart Foundation; MOH = Ministry of Health; IECS = Institute of Clinical and Health Effectiveness; IDEC = Brazilian Institute for Consumer Protection; Various = published by the National Coalition to Prevent Obesity in Children and Adolescents representing a network of civil society organizations in Argentina

Generating evidence was also key to influencing the design of the regulation. As discussions in both the Executive and Legislative branches began to gain momentum, industry and associated stakeholders sought to influence the design of the proposed regulation in ways that would be more favorable to corporate interests, such as the type of FOP labelling scheme and the NPM to be adopted. In 2017, for example, COPAL released a proposal calling for the adoption of a label following the Guideline Daily Amount (GDA) model used in the United Kingdom [112], which has been demonstrated in studies to be less effective than warning labels in shaping consumer understanding, attitudes, and choices [67, 69]. Similarly, the industry argued for a NPM that defined excess nutrients on a ‘per gram’ basis [113], which would have made it easier for companies to evade the labels than with the PAHO NPM. To counter these instances of CPA in the policy process, advocates pointed to the importance of *locally* generated evidence to support arguments for the proposed policy design:

*“What was one of the arguments of the industry? “Okay*,* okay*,* how do we know that the*,* let’s say*,* this front labelling with this black octagon*,* is the most effective for Argentina? Because this has been effective in Chile*,* but how do we know if it is effective in Argentina?” So*,* we have evidence*,* we have scientific evidence*,* but this scientific evidence has not been validated here.” [Advocate*,* Academia]*.

As such, both the Ministry of Health and FIC conducted studies to compare the performance of different labelling schemes amongst the Argentine population [68–70], as well as to demonstrate that the proposed PAHO NPM was in the greatest accordance with the dietary guidelines for the Argentine population compared to other models [71, 72]. One participant pointed to the importance of involving multiple organizations in the generation of evidence to support effective policy design:

*“Another strategy was to work hand to hand with the Ministry of Health*,*and we agreed on what evidence we have to produce*,* at the same time*,* both of us: to have a study from the Ministry of Health that says that the octagon warning level was the best*,* and another study*,* that says the same*,* but from the Civil Society. So*,* it is not only the Civil Society that has this evidence*,* but the Ministry of Health*,* too. And the same with the nutrient profile system.” [Advocate*,* Civil Society]*.

### Building technical literacy

This tenet of instrumental power refers both to the building of technical literacy amongst advocates themselves and amongst decision-makers. Regarding the former, advocates synthesized knowledge on key aspects of the policy process, such as the legal and political landscape surrounding the law. For instance, advocates in civil society led a series of analyses on legal aspects that would influence the policy process, particularly through a collaborative regional study with other countries in MERCOSUR. This included a mapping of the national regulatory framework on FOP labelling [114], an analysis of legal barriers and facilitators to FOP labelling [115], and a report on legal lessons learned from the precedent of tobacco control [116]. Civil society advocates also worked to consolidate knowledge of the political landscape, such as by mapping decision-makers in Congress to understand their stances and guide targeted advocacy:

“*One of our main strategies was to monitor and study the members of the Senate and the Chamber of Deputies; to identify how much power of decision they had within their commissions…. We also wanted to identify who our champions were going to be*,* also the ones who were never going to agree to this law*,* and the ones we could be able to sway in our favor. So*,* we mostly focused on those we could convince*,* and that’s when we asked legislators to have meetings with them and their advisors.” [Advocate*,* Civil Society]*.

Conducting targeted advocacy efforts, such as through one-on-one meetings with key legislators and/or their advisors, was used as a strategy to build technical literacy on the bill in Congress. This proved to be particularly important in the face of CPA that targeted technical aspects of the law. For example, resistance during the later phases of the legislative debate did not oppose the law itself, but rather focused on the need for ‘modifications’ to the text of the law, which, if heeded, would have stalled the passage of the bill. This was particularly the case with Article 6, which established the PAHO NPM as the foundation for the adoption of FOP warning labels and was highlighted by industry stakeholders as a system that would unfairly affect their products [117]. In this context, advocates described the importance of holding meetings with legislators to clarify key concepts:

*“We talked to legislators and advisors; we explained why the bill was written the way it was written; that we must use PAHO’s nutrient profile system; that the industry kept insisting on using a different profile system. You can base the law on unlimited profiles and the law would end up a mess. So*,* we explained to them the importance of each and every article of the law*,* that the law must be approved unchanged.” [Advocate*,* Professional Nutrition Organization]*.

In addition, conducting targeted advocacy was identified as an important approach to advocacy in the context of unequal resources:

*“….they [the industry] knew that they had to engage with all political actors*,* not just “some”. On the other hand*,* NGOs and civil society have fewer resources*,* so they concentrated their relationships with key stakeholders.” [Advocate*,* Advisor to Legislator]*.

### Exposing conflicts of interest and harnessing public pressure

Another key population that advocates sought to influence was the public, accomplished through the strategic use of communication channels. For example, advocates described using traditional and social media to expose industry tactics and encourage accountability of decision-makers, including ‘naming and shaming’ those who had conflicts of interest. Other identified strategies to harness public pressure included conducting communication campaigns with national coverage in public spaces, radio, digital and print media, such as the, “Don’t let them cover your eyes,” (“Que no te tapen los ojos”) [118] campaign, and making use of a digital platform, ‘Activá el Congreso’ [119], which enabled individuals to write directly to legislators. The involvement of advocates who could more effectively reach the public, such as journalists, influencers, youth activist groups and celebrities, was vital to harnessing public pressure. Bringing the debate surrounding the law into the public domain was noted as a key strategy to counter imbalances of power through the policy process:

*“…one more thing about this imbalance is that it is only possible to restore it if civil society plays a very aggressive role on the internet*,* in the media*,* employing certain communication strategies….If the discussion had only taken place within the Chambers*,* we probably would have lost the case.” [Advocate*,* Legislator]*.

Leveraging public pressure proved particularly critical at junctures in the legislative debate where it seemed that the bill would not successfully advance due to interference. For example, following the half-sanction of the bill in the Senate, the bill was assigned for consideration by an unusually high number of commissions within the Chamber of Deputies [120], a strategy advocates identified as one motivated by conflicts of interest held by the president of the Chamber of Deputies to hinder the passage of the bill. Here, one advocate stressed the importance of public pressure in overcoming this obstacle:

*“that’s when Civil Society launched campaigns on Twitter denouncing the number of committees that the bill was assigned to; that made [the president of the Chamber of Deputies]*,* who in another context would not have changed his mind*,* feel singled out and decide to reduce the number of committees to three*,* although they finally ended up being four.” [Advocate*,* Legislator]*.

Once the commissions in the Chamber of Deputies issued a positive opinion on the bill, the final vote in the Chamber of Deputies remained as the final step to adopt the bill. Advocates described another instance at this point in the process where political factors almost prevented the passage of the law, in which one of the two major political parties did not present with a quorum at the session in which the law was to be put to a vote, placing the bill at risk of losing parliamentary status if not approved before the end of the year [121]. Again, advocates pointed here to the importance of public pressure to overcome this obstacle:

*“Another enabling factor appears when society starts to personally start caring about the law. This was very apparent when the Chamber of Deputies didn’t reach a quorum on the bill… then began to circulate a very strong campaign in social media*,* where the public would call out these people and say*,* “How come they don’t want to vote on a bill that involves the health of the people?”. To me*,* that was a very compelling moment*,* I didn’t know that the bill had affected society in this way.” [Advocate*,* Professional Nutrition Organization]*.

### Discursive power

#### Generating counterarguments

Over the course of roughly a year from when the bill was first proposed in 2020 to its passage in 2021, the topic of FOP labelling transformed from a relatively niche and technical topic predominantly discussed within institutional spaces, to one of great political and public interest, with its own hashtag (#EtiquetadoClaroYA) on social media. This transformation reflects a shift in the dominant discourse surrounding the implications of the law for society. Advocates worked to shape the discourse surrounding the law, which required that they be poised to counter a range of economic, technical, legal, and ethical arguments made by the industry and associated stakeholders throughout the policy process (see Table 3 for a summary of key arguments and counterarguments). The knowledge and evidence generated by advocates, as previously detailed in Table 2, played a key role in supporting several of these counterarguments.

**Table 3:** Common arguments used to oppose the proposed regulation and advocate counterarguments through the policy process of the Promotion of Healthy Eating Law in Argentina [122–124]

Type of argument	Argument	Counterargument
	***The proposed regulation…***	
Economic	…will cause job losses and low wages in the food sector.	In Chile, minimal negative impacts have been observed regarding industry employment with the adoption of FOP labelling.
	…will generate additional costs for the sector of the food industry.	The food industry has the resources to adopt the measures without suffering significant economic impacts.
	…will negatively affect the Argentine sugar sector.	Sugar is mostly produced for biofuels, which would not be impacted by the law.
	…will reduce sales.	Food companies generally have a portfolio of different products, some with and some without labels. Companies can also reformulate.
	…will harm companies by prohibiting them from advertising their products.	The regulation does not prohibit all advertising. In addition, it is an opportunity for companies to advertise the absence of seals for a competitive advantage.
	…will disproportionately punish small- and medium-sized enterprises (SMEs).	The law contemplates deadlines for major adaptations with the possibility of extension for SMEs.
Legal	…is not legal because it would not be standardized across MERCOSUR countries.	The economic bloc of MERCOSUR recognizes the States Parties the right to legislate for the protection of the public health of its citizens. Other countries in the bloc have introduced FOP labels at the national level.
	…is not legal because it would not align with WTO standards.	WTO recognizes the right of States to legislate and take measures that they deem necessary to protect public health. See the precedent of tobacco.
	…is not legal because it contradicts provisions of the Codex Alimentarius.	Codex Alimentarius guidelines constitute a minimum floor on which to advance in terms of public policies, but not a limit.
	…will introduce barriers to free trade due to differing packaging requirements.	The regulation applies only to Argentina and would not affect products exported to other countries.
	…will harm the export of Argentine food by creating barriers to international trade	Provisions under the WTO TBT agreement would ensure that the labelling regulation would not introduce undue barriers to trade.
	…contravenes the Argentine Food Code because it will present false information about the real nutrient content of food.	The use of the PAHO NPM and warning labels has been shown to be the most effective at communicating the nutrient content of food and would enhance transparency rather than hinder it.
	…will violate intellectual property	This has been refuted through the precedent of tobacco and UPF regulation in Chile, where such lawsuits have been dismissed.
Technical/ scientific	…has not been shown to decrease overweight or obesity rates.	A period of ample time is needed to observe public health impacts; The motivation for the law should remain consistent with its objective, which is to offer people timely, clear, accurate and true information that enable healthier consumption choices.
	…does not address the root problem of poor diets, which are based on individual choices.	There is ample evidence to support the role of UPF consumption as the root cause of obesity epidemic.
	…has no empirical evidence to show that it will change consumer choices.	Empirical evidence was collected in Argentina demonstrating that the warning label had the highest impact on consumer intention to purchase.
	…uses a NPM with no empirical evidence behind it and is against the dietary guidelines in Argentina.	A comparison of eight nutrient profile systems found that the PAHO nutrient profile demonstrated the highest accordance with the dietary guidelines in Argentina.
	…uses a NPM that does not promote reformulation.	The aim of the labels is not to encourage reformulation, but to inform consumers. However, evidence from Mexico demonstrates the potential for reformulation.
	…will result in over 90% of products being labelled, completely overwhelming consumers.	The law applies only to UPFs, which do not encompass such a high percentage of foods sold in retail settings.
Ethical/social	…misrepresents the nutritional value of certain products.	The label’s use depends on the chemical composition of each product.
	…demonizes packaged food.	The law seeks to protect consumers’ right to information, not to demonize.
	…is a law for rich people/the first world in a context of economic decline and rising food insecurity.	Consumption of UPFs carries disproportionately negative health and economic ramifications for the most vulnerable sectors of the population, and thus is a high priority in this context.
	…will prevent the free delivery of products containing at least one label, preventing donation of food to vulnerable populations in the context of rising food insecurity.	The law will not prohibit the donation of products without warning labels, which would be better for the health of the most vulnerable sectors of the population.
	…confuses consumers and therefore harms individual freedom of choice.	This law upholds the consumer right to transparent information, thereby better enabling freedom of choice, particularly in the context of misleading marketing practices.
	…is not the appropriate approach to shift diets. Education is needed for better choices.	Education and campaigns are important components and should be part of a comprehensive policy to improve food environments. Campaigns are not substitutes to labels, but complements.

Acronyms: MERCOSUR = Southern Common Market, WTO = World Trade Organization; TBT = Technical Barriers to Trade Agreement, PAHO = Pan-American Health Organization, UPFs = Ultra-Processed Foods

Advocates noted that economic arguments, particularly given the context of economic instability and decline in Argentina, gained the most traction against the law.

*“…the strongest argument in a country in Latin America is about*,* we are going to lose jobs*,* employment is going to be affected*,* which is also an argument that the industry is going to go broke*,* that the industry cannot endure this… I mean*,* the rest is more debatable*,* but the economic factor is important and that is where the industry went.” [Advocate*,* International Development Agency]*.

In this context, emerging evidence from Chile demonstrating that neither aggregate employment nor average real wages were affected by food labelling regulation helped support advocates to address these concerns in Argentina [125–127]. Advocates also spoke to the importance of emphasizing the economic ramifications of not acting in the form of rising healthcare costs.

#### Rights-based framing

To address ethical arguments, framing was key. Namely, advocates spoke to the importance of framing the law in communication with decision-makers not just within the paradigm of public health, but other values, such as the right of consumers to transparent information regarding the content of their food:

*“…that was very important*,* to focus on the consumers’ right*,* not focusing only on that eating better was important*,* but you have to know what you are eating*,* then you decide*,* no? And that was very important to convince the legislators.” [Advocate*,* Civil Society]*.

This rights-based framing was also important in harnessing public support, and was pursued in communication campaigns led by civil society, such as the aforementioned “Don’t let them cover your eyes,” (“Que no te tapen los ojos”) campaign, with the slogan, “It is our right to know if a food has excess fat, sugar and/or sodium” [118]. This framing helped to turn the commonly used argument by the industry of individual responsibility and choice on its head by presenting the labels as a tool to enhance individual autonomy rather than hinder it. Another key framing for the law that fostered public support was the protection of vulnerable populations, particularly children and adolescents, from deceptive industry practices:

*“I think it was a combination of different narratives*,* but I would say that the need to protect children and vulnerable social groups was what penetrated the most through society and*,* also*,* the industry’s lies…All of this sparked interest and a feeling of alarm at the same time.” [Advocate*,* Civil Society]*.

Extending narratives beyond the confines of a nutritional perspective to align the framing of the law with the priority values of different movements also brought more advocates into the fold, such as consumer organizations and environmental activists, reaching a broader audience over time.

### Reputation management

The credibility that advocates carried in the arguments they made hinged on their reputation. As such, reputation management was an important aspect of advocates’ work throughout the policy process. For some organizations, particularly those comprised of nutrition professionals, managing internal conflicts of interest was critical. This was the case with the Argentine Federation of Graduates in Nutrition (FAGRAN), an umbrella organization of the Colleges and Associations of Graduates in Nutrition. The work done by the organization to reach an organizational position free of conflicts of interest, spearheaded by a change in leadership in 2018, was a critical factor in providing a degree of legitimacy to the voice of these advocates coming from the field of nutrition. The ability of organizations like FAGRAN to position themselves as entities free of conflicts of interest became a useful tool to spotlight those entities who could not do the same:

*“…we told everyone who we were and we told them that we had no conflict of interest. And when you work on the law*,* people are more willing to listen to what you are saying. On the other hand*,* whenever we would hear arguments against the law*,* we knew those people and organizations always had conflict of interest. So*,* we requested that everyone who participated in the discussion must state whether they had any conflict of interest or not*,* and if they did*,* then what kind of conflict of interest it was.” [Advocate*,* Professional Nutrition Organization]*.

#### Outcomes of power: reflections on policy decision

The law was seen as a key milestone in a broader effort to regulate the widespread availability of UPFs and promote healthier food environments. Advocates spoke particularly of the importance of adopting mandatory FOP warning labels, which define the parameters of products that should be targeted by future regulations. As one advocate explained:

*“…these labels are a gateway to other regulations. That is*,* everything works together: environment*,* advertising*,* sponsorship*,* labelling and taxes. Increasing taxes on sugary drinks. But the gateway is FOP labelling. It will make it easier to discuss a tax when the product has three labels” [Advocate*,* International Development Agency]*.

Advocates also spoke to the importance of the fact that the law included not only FOP labels, but other components that work synergistically to promote healthier food environments:

*“I think that one of the greatest advantages is that you have in a piece of regulation a lot of – you are able to regulate a lot of the aspects included in what should be a healthier food environment. And that’s also something coming from tobacco. When you regulate and you have a tobacco control law*,* it’s not only about regulating the environment. It’s also about regulating the cigarette package. It’s also about regulating the promotion and advertising and sponsorship of tobacco products. So*,* this was a similar rationale.” [Advocate*,* Civil Society]*.

Ultimately, despite pervasive attempts to prevent or hinder the passage of the law, as well as undermine its scope, the law was passed in accordance with the recommendations made by advocates throughout the policy process. Though advocates pointed to many challenges regarding the road ahead for successful implementation of the law, this milestone was regarded as an important success:

*“We enacted the exact law we wanted to enact. I thought it was going to be modified. Because sometimes they propose a good bill and it ends up as a weak law. Ours was whole. It was complete.” [Advocate*,* International Development Agency]*.

## Discussion

This paper examines how advocates were able to harness and exercise structural, instrumental and discursive power to guide the adoption of a regulation on the labelling, marketing and sale of UPFs. Several lessons can be learned from the insights shared by advocates on the Argentine experience on UPF regulation.

First, advocates must take pages from the ‘corporate playbook’ to effectively counter it, particularly to garner structural power. Corporations often pool political, financial and technical resources to undermine public health action, such as through the activities of umbrella entities like COPAL in Argentina. Capacity building to cultivate a collective voice of advocates to promote public health policy decisions is vital [128]. Through informal alliances and formal coalitions that united organizations across the country, advocates in Argentina were able to collectively access discussion spaces, make strategic use of limited resources, cultivate a unified narrative in their arguments and demands, and harness the diverse expertise needed to counter industry interference and effectively reach both decision-makers and the public. Some of the skillsets of those who worked to advocate for the law in Argentina, such as legal experts, trade analysts, political strategists, and communications specialists, have historically been considered outside the purview of public health, despite being vital to achieving meaningful improvements in health policy [129]. Capacity building initiatives to train, recruit and integrate these skillsets into public health efforts are therefore critical to strengthening the ‘public health playbook’ against corporate power in public health policy decisions [4, 59]. In Argentina and in other public health regulatory policy processes worldwide [44, 45, 48, 50, 130], support from international health groups is an important enabler of capacity building. United Nations (UN) agencies play an important role. In Argentina, for example, PAHO and UNICEF took on many key capacity building roles throughout the process, including convening multi-sectoral discussion spaces for agenda-setting and providing financial and technical support for research, advocacy, and communications in support of the law. Advocates also took a page from the corporate playbook by cultivating structural power through the use of ‘revolving doors,’ where several policy champions either moved between or worked across different roles within civil society organizations, academia, professional organizations, national Ministries, and/or international development agencies throughout the policy process, bringing their knowledge, expertise, and networks with them.

Advocates wielded instrumental power by amassing an armada of evidence - localized to the Argentine population, free of conflicts of interest, and corroborated by both the public sector and civil society - to support the rationale for and robust design of the law. While this degree of scientific output is certainly a testament to the extent of advocates’ work to support the policy process, it also alludes to a key challenge to meaningful health policy change in the face of corporate influence: an over-reliance on a strict evidence-based approach [80, 131]. In other contexts, particularly those with limited capacity to conduct health policy research, consolidating such a wealth of localized evidence may very well not be possible. Fostering international communities of practice in which advocates and decision-makers can exchange the knowledge and evidence cultivated from the experience of other countries that have successfully advanced on adopting UPF regulation, as was also done through regional knowledge exchange facilitated by advocates in Argentina, will be important. As demonstrated in the precedent of tobacco control [45, 132], international networks of advocates can also play an important role in defending national regulations against corporate attempts to undermine them.

Several lessons can also be gleaned from Argentina’s experience cultivating discursive power in the context of this law, particularly on the implications of FOP warning labels. For one, advocates emphasized the importance of framing the labels as autonomy-enhancing tools that provide transparent information as key to building support amongst decision-makers and the public. This framing is particularly important in the context of prevailing neoliberal paradigms that purport the importance of individual responsibility and autonomy in decision-making, which are often exploited by industry actors to undermine regulation [131, 133], as it positioned the law as one that would better enable individual autonomy rather than limit it. Previous literature has demonstrated a limited commitment from both national governments in Latin America and international health groups to adopting a rights-based discourse for UPF regulation [10], indicating an important area for improvement. In addition to this advantage with framing, advocates also understood that mandatory FOP warning labels provided an important foundation for comprehensive UPF regulation by delineating the health-harming commodity that must be regulated. This is particularly important in the context of UPFs where, unlike in the case of tobacco, for example, industry stakeholders can more readily argue health benefits associated with UPFs, in some cases even adding beneficial micronutrients as a tactic to resist regulatory approaches [134]. Together, these insights suggest that pursuing the adoption of mandatory FOP warning labels could be an important foundation for pursuing other important regulations to improve the healthfulness of food environments. Indeed, in Argentina, FOP warning labels were adopted as the foundation of a suite of reinforcing measures that also prohibited the marketing, donation and sales of labelled products in particular settings, including schools and social support programs. FOP warning labels could also support the introduction of additional priority policies to limit the availability and affordability of labelled UPFs and promote that of healthy, minimally processed food products, such as taxes and subsidies [134]. The potential of FOP warning labels to open the door to additional regulation could also be applicable in other contexts; however, additional research, such as that which examines the influence of the order in which policy measures are introduced on political and public support for climate policy [135–137], is needed to examine the generalizability of this strategy outside the Argentine context. In addition, as in Argentina and other cases [138, 139], it must be noted that industry stakeholders can still capitalize on neoliberal narratives of individual responsibility to try and undermine FOP warning labels, despite their function as an information-provision tool.

Besides cultivating greater support amongst decision-makers and the public, advocates observed that expanding the need for UPF regulation beyond the confines of a public health narrative also brought additional advocates into the fold in Argentina, such as consumer associations, youth activists, and influencers, all of which contributed to expanding support for the law. By the late stages of the legislative process, the law was seen by many as not only a matter of public health, but one of protecting human rights, safeguarding against corporate control and political corruption, and fostering a more equitable society. This experience in Argentina corroborates a key strategy that has been identified as vital to building a ‘public health playbook,’ against modern corporate power in public health policymaking: linking with other social movements to cultivate collective solidarity [59]. This is certainly the case for climate and sustainability activism, for example, which continues to garner strong civic engagement worldwide [140]. The connections between the production and consumption of UPFs and environmental outcomes must be strengthened in research, in advocacy, and in policy decisions [141]. Other discursive strategies employed in Argentina further corroborate those that have been identified as vital to building a ‘public health playbook,’ including the importance of debunking corporate arguments, of exposing industry tactics to the public, and of leading by example against conflicts of interest by developing rigorous standards against them within public health organizations [59].

This study is subject to a few limitations. First, this paper focuses on a single case study, limiting the generalizability of the findings to other contexts. For instance, advocates in Argentina noted that they were readily invited alongside other stakeholders, including food and beverage industry actors, to speak within relevant spaces in the Legislative and Executive branch throughout the policy process. This was not the case in Mexico, for example, where civil society actors were excluded from formal participation in relevant institutional discussion spaces for advancing on UPF regulation, reflecting a long legacy of elite-based health policymaking [55]. In general, Argentina has a strong legacy of civil society activities and activism related to health policy issues as demonstrated in the precedent of tobacco control [142] and reproductive rights [143], for example, making it difficult to compare the power dynamics of the policy process to one in which civil society actors have played a different role in health policymaking to date. Another limitation is the retrospective examination of a successful policy decision, which may have introduced bias regarding the importance of certain strategies and enablers. We attempted to mitigate this bias by triangulating information from interviews with that synthesized from a review of media articles, press releases, and reports written leading up to the passage of the law. In addition, we had limited access to stakeholders who participated in certain spaces where the policy was discussed, limiting our insights into power dynamics at play in these spaces. For instance, a burgeoning area of research examines how international trade agreements are being leveraged to hinder nutrition policy action [75–80]. Several advocates spoke broadly to the challenge of an alternative proposal to reach a regional agreement on FOP labelling through MERCOSUR; however, we were not able to explore the influence of corporate power, or strategies to counter it, at this level of governance in depth due to limited access to stakeholders involved in those discussions. The influence of the politicized nature of the topic must also be acknowledged here. Namely, in certain instances, participants noted that they could not be completely forthcoming with their experience for fear of political backlash, though these instances were limited in number and scope. Consolidating lessons learned from advocate experiences addressing corporate power in supra-national food governance, as in the case of tobacco [77, 144], thus constitutes an important area of future research. Finally, lessons learned from the Argentinian experience should not remain static. Future examinations of power dynamics in this case, or others, should encompass the period of policy implementation, which was highlighted by participants as a formidable challenge in the face of continued corporate attempts to undermine it following its adoption. These challenges may have since been compounded with the election of a far-right libertarian president in November 2023, providing a context in which to examine how advocates leverage power, particularly discursive power, in an administration characterized by a prevailing neoliberal ideology.

## Conclusions

The use of corporate power to undermine UPF regulatory decisions is increasingly well-documented; however, analyses of how power can be leveraged to promote successful policy decisions are scant. Learning from the small precedent of countries that have managed to successfully adopt robust regulation on UPFs is an important opportunity to strengthen this knowledge in pursuit of a ‘public health playbook’ against corporate power. Leveraging a framework designed to analyze the role of power in public health policymaking, we demonstrate how advocates wielded structural, instrumental, and discursive power to support the passage of the Promotion of Healthy Eating Law in (Ley 27,642) in Argentina. The experience of advocates in Argentina carries important lessons that may be applicable to other countries looking to advance on the topic, including the importance of cultivating a collective movement in support of regulation, the need for synthesis of knowledge and evidence to weather corporate interference, and the promise of shaping dominant discourse so as to better reach both decision-makers and the public.

## Electronic supplementary material

Below is the link to the electronic supplementary material.

Supplementary Material 1

## Data Availability

The datasets generated and/or analyzed during the current study are not publicly available due to them containing information that could compromise research participant privacy/consent, but are available, with restrictions applied, from the corresponding author on reasonable request.
