# Supplementary material for: How can advocates leverage power to advance comprehensive regulation on ultra-processed foods? learning from advocate experience in Argentina
Source: Global Health. 2024 Sep 9;20:68. doi: 10.1186/s12992-024-01069-1 (PMC11385800; doi:10.1186/s12992-024-01069-1)
Supplement: Supplementary file 1 — Supplementary Material 1 [file 12992_2024_1069_MOESM1_ESM.docx]

**How can advocates leverage power to advance comprehensive regulation on ultra-processed foods? Learning from advocate experience in Argentina**

# **Annex 1. Document Review**

**Table A1.** Number of media articles related to the policy process for Ley 27,642 reviewed, by media outlet

| Source | | Description^*^ | Number of Articles |
| --- | --- | --- | --- |
| Press | *Clarín* | Popular daily | 54 |
|  | *La Nación* | Respected conservative daily | 47 |
|  | *Crónica* | Tabloid daily | 16 |
|  | *El Cronista* | Business | 19 |
|  | *La Prensa* | Argentina’s oldest newspaper | 4 |
|  | *Pagina 12* | Left-wing daily | 13 |
| News agencies/ internet | *Télam* | State-run | 50 |
|  | *Noticias Argentinas* | Set up by privately-owned newspapers | 21 |
|  | *Infobae* | News portal | 45 |
| Total | | | **269** |

*According to BBC Media Guide to Argentina. Available at <https://www.bbc.com/news/world-latin-america-18707517>

**Table A2.** Number of press releases and reports related to the policy process for Ley 27,642 reviewed, by stakeholder organization website searched.

| Stakeholder Org. | | Press Releases | Reports | Total |
| --- | --- | --- | --- | --- |
| Public sector | *Gobierno Argentina* | 12 | 0 | 12 |
|  | *Ministerio de Salud* | 0 | 1 | 1 |
| Civil society | *FIC* | 24 | 1 | 25 |
|  | *FUNDEPS* | 4 | 4 | 8 |
|  | *FAGRAN* | 18 | 4 | 22 |
| International development agencies | *PAHO Argentina* | 16 | 1 | 17 |
|  | *UNICEF Argentina* | 10 | 10 | 20 |
|  |  |  | **Total** | **105** |

**Table A3.** Summary of documents reviewed, by year they were published.

| Document Type | Year of Publication | | | | | | Total |
| --- | --- | --- | --- | --- | --- | --- | --- |
|  | **2016** | **2017** | **2018** | **2019** | **2020** | **2021** |  |
| Media articles | 2 | 2 | 7 | 4 | 41 | 213 | 269 |
| Press releases | 2 | 3 | 9 | 14 | 25 | 30 | 83 |
| Reports | 1 | 1 | 1 | 4 | 10 | 5 | 22 |
| Total | | | | | | | 374 |

**Table A4.** Major milestones in the policy process leading up the adoption of the Promotion of Healthy Eating law.

| Period | Date | Milestone |
| --- | --- | --- |
| Agenda setting | Feb 8, 2016 | PAHO publishes a nutrient profile model to define critical limits for sugar, salt, and fat in ultra-processed foods. |
|  | May 27, 2016 | Chile’s law on food labelling and advertising (Ley 20,606) comes into force. |
|  | Sep 6, 2016 | Argentine government announces the creation of the National Healthy Eating and Obesity Prevention Program in the Ministry of Health, with plans to establish agreements with the food industry on composition, labeling and marketing. |
|  | June 2017 - 2020 | PAHO and UNICEF begin to organize a series of meetings inviting key champions from successful policy precedents in the region, beginning with Senator Gilardi from Chile and continuing with stakeholders from Peru, Uruguay and Mexico. Several stakeholders are convened, including civil society and legislators in Congress. |
|  | June 2017 | At an event organized by PAHO, UNICEF, and directorates of the Chamber of Deputies, Argentina approves the five-year Action Plan for the Prevention of Obesity in Children and Adolescents, which established four axes of regulation. |
|  | June 2018 | Alongside other countries in MERCOSUR, the Ministry of Health signs an agreement with other countries to promote the FOP labelling in Argentina. |
|  | Aug 2018 | Officials, legislators, experts, academics, and civil society leaders call for the establishment of FOP labeling in Argentina during a conference held at the National Congress by the PAHO and UNICEF and the Chamber of Deputies. [event: the Conference on Consumer Rights, Front Labeling of Foods and Health]. |
|  | Sep 2018 | The president of Uruguay signs a decree to establish front of package labelling. |
|  | Sep 2018 | The Special Rapporteur on the right to food, Hilal Elver, visits Argentina, pointing out that the standards on labeling and nutritional information do not comply with international recommendations. |
|  | May 2019 | First meeting on Frontal Labelling of Food and Beverage products is held in MERCOSUR, looking to move forward on the framework signed by health ministers in 2018 on FOP labelling. |
|  | July 2019 | Conference on Obesity Prevention held this week in the Chamber of Deputies of the Nation, carried out by PAHO, UNICEF, the General Directorate of Parliamentary Diplomacy of Congress and the Observatory of Human Rights of the Senate. Uruguayan experience presented. Several legislators also present bills to incorporate regulations that help prevent obesity, such as front labeling, limiting advertising directed at children and protecting school environments. |
|  | August 5, 2019 | A declaration signed by more than 100 organizations and leaders in the field of health was published, where they request that the governments of Latin America enact effective policies for front labeling of warnings to promote the right to information, health and proper nutrition. |
|  | Oct 27, 2019 | Alberto Fernández of the center-left Peronist Frente de Todos (Front for All) coalition defeated current President Mauricio Macri of the center-right Juntos por el Cambio. |
| Legislative passage - Senate | Aug 2020 | Minister of Health holds a working meeting with the national ministers of Productive Development, and of Agriculture, Livestock and Fisheries, in which they harmonized the work and the inter-ministerial coordination that concluded in the presentation of a project for frontal nutritional labeling of food. Plans are made to present at next meeting of CONAL in September |
|  | Oct 2020 | The bill is given a joint positive opinion from the Health and Industry Commissions of the Senate. |
|  | Oct 2020 | The Ministry of Health ratifies its commitment to advance policies against obesity and overweight during the Conference on childhood obesity and overweight, in which the author of Mexico's front food labeling law presented the new regulations of that country. National legislators also participate when the Senate is advancing a project to incorporate labeling in the country. |
|  | Oct 2020 | The bill is given half sanction in the Senate (64 in favor, 3 against, no abstentions). |
|  | Oct 2020 | UNICEF, PAHO, and the Food and FAO launch a campaign to ask the sanction of the front food labeling bill that this Thursday was approved by the Senate of the Nation and sent to the Chamber of Deputies. |
| Legislative passage – Chamber of deputies | Nov 2020 | More than 100 scientific, academic, civil society and health organizations from across the Latin American region signed a declaration requesting the National Deputies to approve the food labeling project without delays or changes. |
|  | Mar 4, 2021 | A few days before the start of the Ordinary Sessions, FIC Argentina launches the *#DeFrente* campaign so that the Chamber of Deputies of the Nation approves, without changes or further delays, the bill that seeks to implement a Front labeling on food and beverages. |
|  | Apr 14, 2021 | A meeting of CONAL is held to discuss the FOP labelling project put forth by the Executive branch, which would also go through MERCOSUR. Civil society speaks out against this initiative because it is less robust than the legislative approach. |
|  | Jun 28, 2021 | A communication campaign organized by civil society, *Que no te tapen los ojos,* is published in public, radio, digital and print media to request that the Chamber of Deputies approve, without further delays or changes, the project for front labeling of warnings on food and beverages. |
|  | Jul 13, 2021 | The bill is approved by four internal Commissions (General Legislation; Social Action and Public Health; Consumer Defense User and Competition; Industry) of the Chamber of Deputies. |
|  | Sep 2, 2021 | PAHO and UNICEF host a Conference on the Law for the Promotion of Healthy Eating, aimed at legislators, decision makers of the Executive Branch and civil society, which seeks to generate a contribution from the international perspective to the legislative agenda of front labeling in Argentina, again bringing many guests from other countries to speak on their regulatory experiences. |
|  | Oct 5, 2021 | The bill is supposed to be discussed, but is not due to the lack of a quorum reached amongst the ruling party |
|  | Oct 26, 2021 | The bill is passed by an almost absolute majority (220 votes in favor, 22 against). |

CONAL = National Nutrition Commission; COPAL = Coordinator of Food Product Industries; FAO = Food and Agricultural Organization of the United Nations; FIC = Inter-American Heart Foundation; FOP = front-of-package; MERCOSUR = Southern Common Market; PAHO = Pan-American Health Organization; UNICEF = United Nations Children’s Fund

**Table A5.** Key stakeholders in the policy process leading up to the adoption of the Promotion of Healthy Eating law.

| Type | Stakeholder |
| --- | --- |
| Legislative branch | - National Congress: Senate (Senado), Chamber of Deputies (Camara de Diputados) - Policy champions: Senator Sagasti (Mendoza, Frente de Todos), Senator Cobos (Mendoza, Juntos por el Cambio) |
| Executive branch | - Ministry of Health *(Ministerio de Salud de Argentina)* - Ministry of Agriculture, Livestock and Fishery *(Ministerio de Agricultura, Ganadería y Pesca)* - Ministry of Productive Development *(Ministerio de Desarrollo Productivo – MDP)* - National Food Institute (Instituto Nacional de Alimentos – INAL) - National Agrifood Health and Quality Service (SENASA) - National Administration of Medicines, Food and Medical Technology (ANMAT) |
| International organizations | - United Nations Children's Fund (UNICEF) - Pan-American Health Organization (PAHO) - Food and Agriculture Organization of the United Nations (FAO) |
| Industry and associated organizations | - Coordinator of Food Product Industries (Coordinadora de industrias de Productos Alimenticios – COPAL) - Argentine Sugar Center (Centro Azucarero Argentino - CAA) - United States Chamber of Commerce in Argentina (Cámara de Comercio de los Estados Unidos en Argentina - AmCham Argentina) |
| Civil society | - Interamerican Heart Foundation (Fundacion Interamericana de Corazon – FIC) - Foundation for the Development of Sustainable Policies (Fundación para el Desarrollo de Políticas Sustentables - FUNDEPS) - SANAR Foundation (Fundación SANAR) - Collective Conscious (Consciente Colectivo) - Consumers of Argentina (Consumidores Argentinos) |
| Academia | - Free Chair of Food Sovereignty, University of Buenos Aires (Cátedra Libre de Soberanía Alimentaria – CaLiSA) |
| Professional nutrition association | - Argentinian Federation of Nutritionists (Federación Arg. de Graduados En Nutrición - FAGRAN) |
| Inter-agency coalitions | - National Coalition to Prevent Childhood Obesity (Coalición Nacional para Prevenir la Obesidad Infantil) - Network of Lawyers for Food Sovereignty (Red de Abogadas y Abogados por la Soberanía Alimentaria - REDASA) - Network of Free Chairs of Food Sovereignty and Related Groups (Red de Cátedras Libres de Soberanía Alimentaria y Colectivos Afines – RED CALISA) - Association of Chefs and Businessmen linked to Argentine Gastronomy (Asociación de Cocineros y empresarios ligados a la Gastronomía Argentina – ACELGA) |

# **Annex 2. Interview Guide**

*Thank you for agreeing to participate in this research project. I know your time is very precious, and it is much appreciated that you are spending this time sharing your knowledge to advance this work.*

*The goal of this project is to understand the political challenges that were faced, and ultimately the strategies that were leveraged by policy advocates to promote the advancement of the Promotion of Healthy Eating Law in Argentina. The context for this project is the understanding that this is a very difficult sphere for policy advocates to navigate, as public health policies often face strong opposition from stakeholders with high financial and political power. With this in mind, it is important to learn from the few examples in the world where progress has been made.*

*You have been selected to be interviewed due to your extensive first-hand experience and involvement through the development of the Healthy Eating Promotion Law in Argentina. Over the course of this study, I will speak with several experts, like you, to get a detailed understanding of this topic.*

*Before we start, do you have any questions?*

| Topic | Questions | *Prompts* |
| --- | --- | --- |
| Overview / easing in | As a part of this project, we are considering two phases of the political cycle for the Healthy Eating Promotion Law: I) the agenda-setting phase, which focuses on the period leading up to the proposal of the unified Healthy Eating Promotion bill in 2020; and II) the legislative passage phase (2020-2021), which begins with the initial proposal of the bill through the debates in the Senate and Chamber of Deputies and ends with the official approval of the law.  To start us off, could you please provide an overview of you and your organization’s role in the development of the Healthy Eating Promotion Law (Ley 27,642)? *Please specify as much as possible the timeline and/or evolution of your role relative to the three phases mentioned.*  *Major activities, how they changed through the different phases* | |
| Forms and mechanisms of power | Challenges | |
|  | Could you please provide an overview of challenges that you and your organization encountered while trying to advocate for the advancement of the law? Where you can, please use specific examples.  *Major arguments made against the law, conflicts of interest, etc.*  In your opinion, which challenges were the biggest obstacles to the law's advancement? Why? | |
|  | Strategies | |
|  | What activities did you and/or your organization take part in to try and advance the law?  Did your organization have a clear strategy to address industry resistance? If so, could you please describe the strategy?  *Increasing transparency of conflicts of interest, evidence dissemination, policy framing, etc.*  Which strategies do you feel were most important or successful for countering resistance? | |
| Dimensions of power | Levels – global, national, local | |
|  | Which discussion spaces was your organization involved in through the legislative process of the law to advocate for the law?  *Commissions in National Congress, public-facing platforms,*  *meetings with international agencies, civil society coalition, MERCOSUR, etc.* | |
|  | Spaces - closed, open, invited, claimed | |
|  | Were there spaces where the law was being discussed where you felt your organization or others with a similar stance were excluded from the discussion?  Did you feel in certain instances that your organization had to fight to get ‘a seat at the table’ to present your point of view on the law? If so, could you please provide additional details? | |
| Contextual factors | Which factors do you feel enabled you and your organization to successfully advance your activities to advocate for the advancement of the law? | |
| Lessons learned and closing | If you think of advocates in a similar role to you in another country looking to advance a similar law, what do you think are the most important lessons to draw from Argentina’s experience?  Are there any topics that we have not had the chance to cover that you feel it would be important to discuss? | |
